# Supplementary material for: Face coverings and respiratory tract droplet dispersion
Source: R Soc Open Sci. 2020 Dec 23;7(12):201663. doi: 10.1098/rsos.201663 (PMC7813263; doi:10.1098/rsos.201663)
Supplement: Supplementary Information [file rsos201663supp1.docx]

# Supplementary Information

## Table of Contents

## S1. MaskS TYPES AND MATERIALS

## S2. Laser-Imaging of droplets in flight

## S3. Droplet deposition

## S4. Characterising particle size and velocity

## S1. MaskS TYPES AND MATERIALS

The surgical masks were “Disposable Medical Masks” purchased through the University of Edinburgh’s Pharmacy from Henan Yunda Medical Equipment Co Ltd., 158 Dinzhang Road, Changyuan City, Xinxiang City, Henan Province, China (Certificate of Compliance to EN 148:2001 and A1:2009 Standard, number 0P200315.HYMUT56). The handmade masks were “Reusable Fabric Face Mask, White, One Size” purchased through Amazon (item model number: MSK10UK; ASIN: B08888C81Z) from FM London Accessories, Unit 13, Basset Court, Loake Close, Grange Park, NN45EZ, UK.

To gain further insights into the masks we used in our tests, we sought to obtain micrographs of their plots. To this aim we excised small sections of the masks (roughly 1 cm in width and 2 cm in length) and attached them to a microscopy glass slide to proceed to imaging via phase-contrast micrography. We used a CFI Plan Fluor 4X objective mounted on a Nikon Eclipse Ti inverted microscope, equipped with an Andor Zyla sCMOS camera, resulting in a resolution upon magnification of 1.63 µm/pixel. A field of view of 2.77 mm^2^ in total, was acquired in bright-field using NIS-Elements imaging software. The interwoven cotton threads of hand-made masks (Figure S1A) leave gaps of around 50 µm (white spots). Surgical masks are formed from multiple layers of fibres with a broader spectrum of gap sizes (Figure S1B).

| A  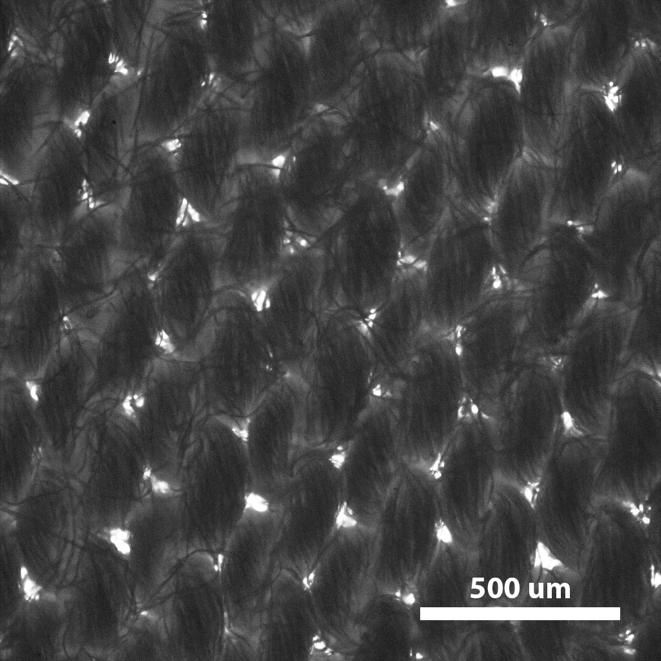 | B  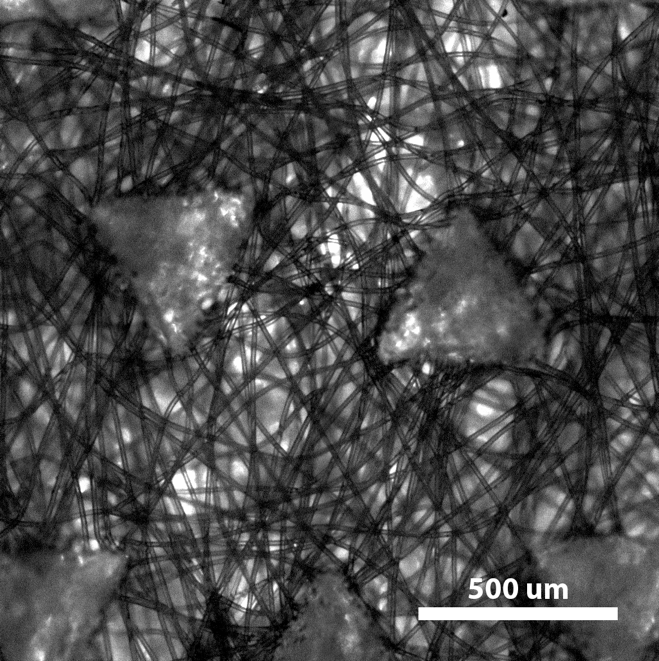 |
| --- | --- |
| Figure S1. Micrographs of Handmade (A) and Surgical Masks (B). The structural properties, and differences, of the plots are clearly visible in these images: brighter spots highlight “pores”, i.e. points where the individual would be exposed to suitably small particles from the outside. | |

## S2. Laser-Imaging of droplets in flight

To image larger respiratory droplets in flight, we projected a thin laser sheet along the vertical plane through the mouth of the manikin and used a photographic camera to capture the light scattered by droplets passing within this plane.

To capture images for analysis, the camera was placed at varying positions from the manikin. Position A (Figure 1A) was located directly in front of the mouth, with its centre at (x, y) = (0.085, -0.027) m, relative to the centre of the mouth. The centre of the other positions (1-7) were at y = -0.32 mm in the vertical direction. The centre of positions 1, 2, 3, 4, 5, 6, and 7 were at x = 0.2 m, 0.34 m, 0.48 m, 0.32 m, 1.16 m, 1.65 m, and 2.15 m in the longitudinal direction, respectively. Positions 1-4 were used for speaking conditions, and positions 2, 4, 5-7 were used for coughing conditions. Fluorescent droplets appeared as segments on the images, with a length proportional to their speed (Figure S2).


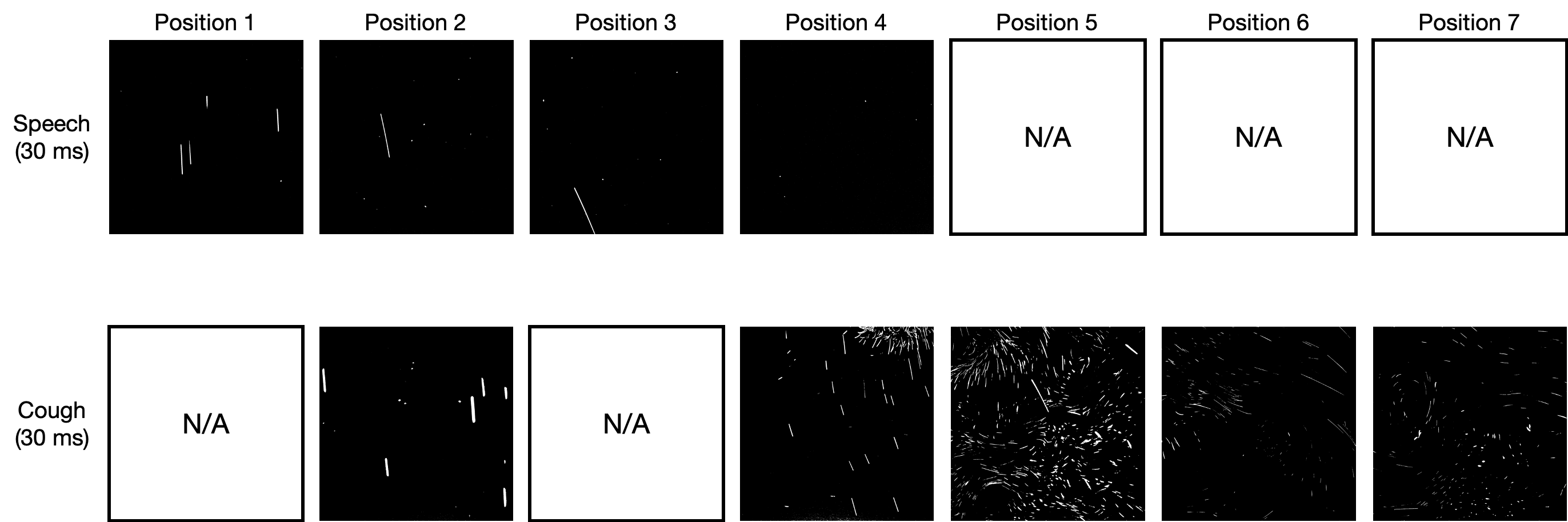


### Figure S2. Examples of In-Flight Droplets. Examples of images captured at positions 1-7 (directly above the table) for speaking (upper row) and coughing (lower row).

## S3. Droplet deposition

To provide an overview of all of the droplets deposited from the manikin, we used UV light to image the distribution of fluorescent particles that accumulated on white paper placed in front of it. An example result is shown in Figure S3A, which shows the droplet distribution observed after a speaking test. We also used microscopy to corroborate the results obtained with UV light imaging. A sample image, acquired on a slide placed 25 cm from the manikin, is shown in Figure S3B.

| A |  |
| --- | --- |
| B | 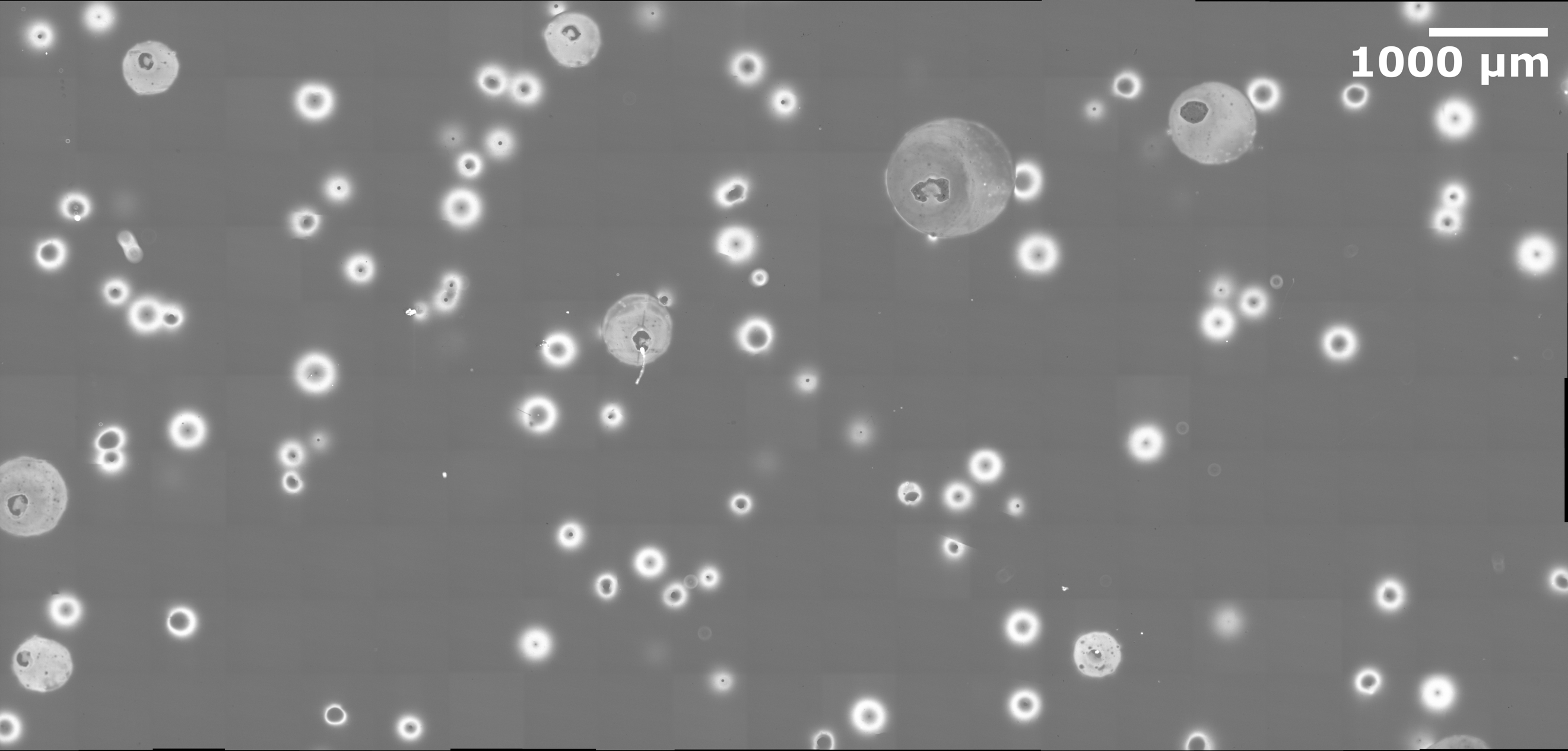 |

### Figure S3. Example of Droplet Distributions on the Table. Droplets found on the table for a speaking test in absence of face covering measured Using UV light imaging (A) and microscopy (B).

## S4. Characterising particle size and velocity

We used shadow imaging to characterise particle size range and behaviour. An 8-bit CCD camera with a resolution of 2056 x 2060 pixels with a Tamron 180 mm F3.5 SP AF Di Macro Lens was used to image a physical plane of 26.96 x 27.01 mm. This gives a resolution of 13.1 μm/pixel, so the following particle diameters are given to ± 7 micron. A fluorescent light was used as a backlight and a light diffuser was used to create a uniform background (Figure S4). Water droplets coloured by black food dye were visualised. Measurements were taken at seven positions: A, B, 2’, 4’, 5’, 6’, and 7’. Position A was located directly in front of the mouth, as for the laser tests, with its centre at (x, y) = (0.085, -0.027) m, relative to the centre of the mouth (Figure 1A). Position B was 0.2 m above position A at (x, y) = (0.085,  0.173) m. The centre of positions 2’, 4’, 5’, 6’, and 7’ were located 45 mm below the centres of the corresponding laser test positions 2, 4, 5, 6 and 7, respectively (y = -0.365 m). Images were recorded with apertures f/3.5, f/8, f/11 and f/16 combined with exposure times of 0.07, 0.5,
1 - 1.5 and 2 ms, respectively.

###
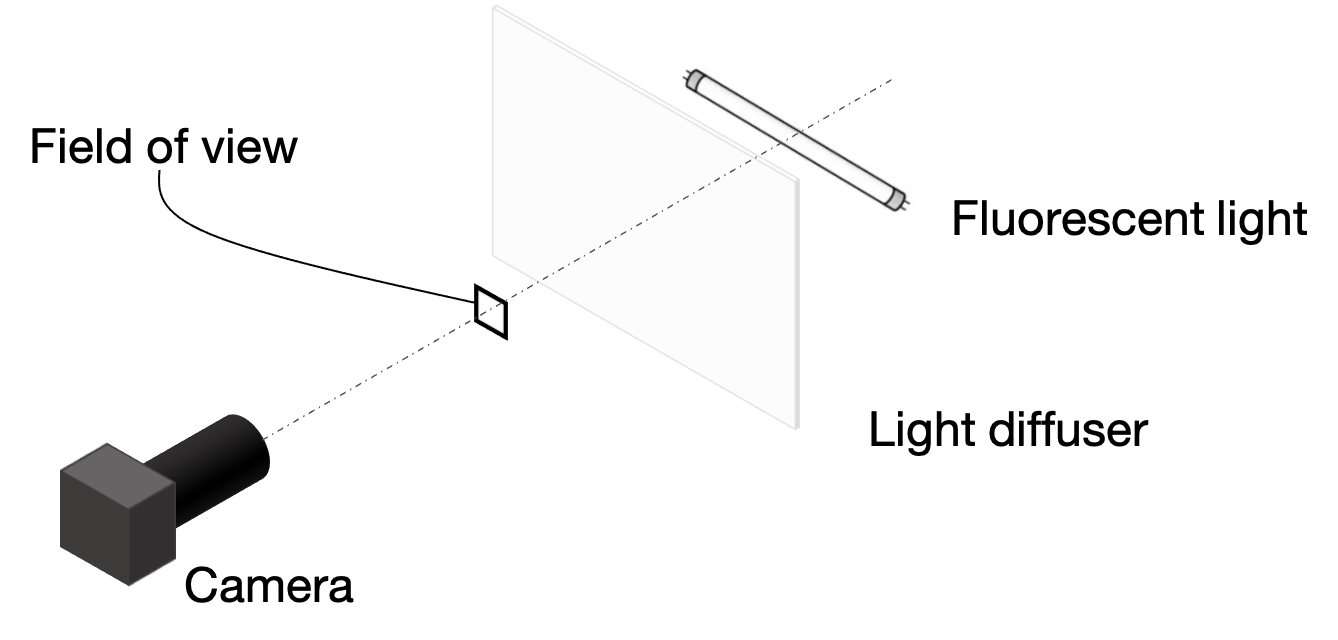


### Figure S4. Experimental Setup for Shadow Imaging.

The black coloured water droplets appeared as segments with thickness equal to their diameter and length proportional to their speed (Figure S5). Overall, the smallest and largest particles seen were 26 ± 7 µm and 590 ± 7 µm, respectively, and the largest particles displaying non-ballistic behaviour were 170.3 ± 7 µm.

Particle behaviour was affected by the background flow to an extent depending on both particle size (smaller particles being more affected) and the strength of the flow. With shadow imaging, we could differentiate between particles behaving ballistically or non-ballistically based on pathline angle (Table S5). Specifically, measured anti-clockwise from the positive x-axis (Figure 1), pathline angles smaller than 0 and less than or equal to -90 degrees were considered to be ballistic, while angles greater than or equal to zero and less than 90 degrees were considered non-ballistic. From the laser imaging using a wider field of view, we could both visualise flow eddies and track some particles between frames, which enabled us to distinguish floating and falling behaviours.

From the above results, we draw the following conclusions. First, we can see particles smaller than 26 ± 7 µm with the laser. Second, we can measure the size of droplets as small as 26 ± 7 µm with shadow imaging and see that these often follow the background flow instead of falling. Third, we can see that droplets up to 170 ± 7 µm sometimes travel non-ballistically and that the larger droplets are only minimally deviated from a ballistic trajectory. Hence, for the laser particle tests, we concluded that we counted all droplets that fell ballistically and that we had a resolution that allowed us to see droplets small enough that they did not fall but followed air currents instead.


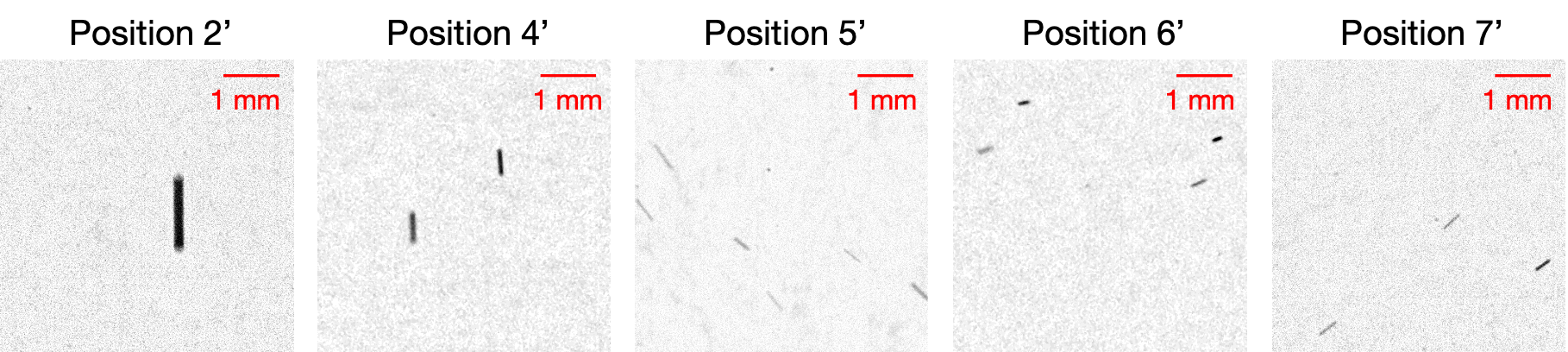


Figure S5. Examples of Shadow Images. Example of images captured at positions 2’, 4’, 5’, 6’, and 7’ using an exposure time of 2 ms. The images are cropped with 5.24 mm x 5.24 mm.

### Table S5: Particle Behaviours and Diameters. All values are ± 7 µm.

| Position | Particle size (µm) | | |
| --- | --- | --- | --- |
|  | Overall | Non-ballistic | Ballistic |
| B | < 26 | < 26 | < 26 |
| A | 26 - 590 | 26 - 144 | 26 - 590 |
| 2 | 39 - 550 | 52 - 170 | 39 - 550 |
| 4 | 26 - 301 | 26 - 144 | 39 - 301 |
| 5 | 26 - 341 | 26 - 92 | 26 - 341 |
| 6 | 26 - 223 | 26 - 144 | 26 - 183 |
| 7 | 26 - 131 | 26 - 52 | 26 - 131 |
